# Pharmacokinetics of butorphanol following intravenous and intramuscular administration in donkeys: A preliminary study

**DOI:** 10.3389/fvets.2022.979794

**Published:** 2022-09-23

**Authors:** Lisa Ebner, Odette O, Bradley Simon, Ignacio Lizarraga, Joe Smith, Sherry Cox

**Affiliations:** ^1^Department of Clinical Sciences, Ross University School of Veterinary Medicine, Basseterre, Saint Kitts and Nevis; ^2^College of Veterinary Medicine, Lincoln Memorial University, Harrogate, TN, United States; ^3^SAGE Veterinary Centers, Dublin, CA, United States; ^4^Department of Small Animal Clinical Sciences, College of Veterinary Medicine and Biomedical Sciences, Texas A&M University, College Station, TX, United States; ^5^Department of Biomedical Sciences, Ross University School of Veterinary Medicine, Basseterre, Saint Kitts and Nevis; ^6^Department of Large Animal Clinical Sciences, College of Veterinary Medicine, University of Tennessee, Knoxville, Knoxville, TN, United States; ^7^Department of Biomedical Sciences, College of Veterinary Medicine, Iowa State University, Ames, IA, United States; ^8^Department of Biomedical and Diagnostic Sciences, College of Veterinary Medicine, University of Tennessee, Knoxville, Knoxville, TN, United States

**Keywords:** butorphanol, opioid, *Equus asinus* L. (donkey), pharmacokinetics, donkey

## Abstract

The pharmacokinetics of butorphanol after intravenous (IVB) and intramuscular (IMB) administration in donkeys were determined in this preliminary study. Healthy male gelded donkeys (*n* = 5), aged 6–12 years old, were administered 0.1 mg/kg butorphanol IV or IM in a randomized, crossover design. Blood samples were obtained at predetermined intervals for 24 h (IVB) and 48 h (IMB) after administration. Plasma butorphanol concentrations were determined by high performance liquid chromatography and pharmacokinetic parameters were calculated. Following IVB administration, mean (± SE) apparent volume of distribution, elimination half-life, total body clearance, and area under the plasma concentration time curve from time 0 to infinity (AUC_0−∞_) were 322 ± 50 mL/kg, 0.83 ± 0.318 h, 400 ± 114 mL/h/kg, 370 ± 131 h·ng/mL, respectively. After IMB administration, a maximum plasma drug concentration of 369 ± 190 ng/mL was reached at 0.48 ± 0.09 h. The IMB AUC_0−∞_ was 410 ± 60 h·ng/mL. Bioavailability of IMB was 133 ± 45%. The pharmacokinetics of butorphanol in healthy donkeys was characterized by faster elimination half-life compared to values from the equine literature.

## Introduction

As of 2018, the worldwide donkey population was estimated to be over 50 million animals, with most serving in the capacity of working animals and essential to the livelihood of people in developing countries (1, 2). However, there is also a growing trend of donkeys kept as pets or used for physical or emotional therapy in children with special needs or to visit the elderly (3). Typically, veterinarians treat donkeys as small horses, even though the majority of drug pharmacokinetic data collected in donkeys shows a difference in metabolism between the species (4–11). Suggested drug dose protocols for horses must be cautiously extrapolated to the donkey and mule patient, along with careful monitoring for any unintended effects (10).

Butorphanol is a synthetic κ-opioid receptor agonist and a μ-opioid receptor antagonist drug that was originally labeled for antitussive purposes in dogs, but is also approved as an analgesic in cats and horses (12). The effects of butorphanol in horses have been studied, specifically to determine the optimal dose (13), the pharmacokinetic effects (13–16), age considerations (17), and effects of exercise (18). The sedative and analgesic effects of detomidine-butorphanol was determined to be an effective combination for standing procedures in donkeys (19), as was the combination of xylazine and butorphanol in donkeys (20). Butorphanol combined with xylazine and ketamine has also been compared to other injectable anesthetic agents in Mammoth asses (21) and mules (22). It is known that the cardiopulmonary effects after administration of butorphanol in horses causes no significant changes in heart rate, mean and diastolic arterial pressure, or cardiac output (23). Butorphanol has been associated with adverse behavioral and gastrointestinal tract effects in horses including ataxia, decreased borborygmi, and decreased defecation (14). To our knowledge, there are no studies determining the pharmacokinetics of butorphanol in donkeys. The specific aim of the study was a preliminary description of the pharmacokinetics of butorphanol in donkeys after intravenous and intramuscular administration.

## Materials and methods

### Animals

Eight adult, male-castrated, university-owned healthy donkeys (*Equus asinus*) weighing 146.7 ± 12.9 kg, aged 8.2 ± 1.6 years, with body condition score 3.8 ± 0.5 (out of 5), respectively were enrolled in this study, but only five donkeys (*n* = 5) were able to be used for cross-over comparison.

They were determined to be healthy by means of physical examination, complete blood count and serum biochemistry. The donkeys were kept as a herd within a grass pasture and were also provided local Guinea grass twice a day and water *ad libitum*. Food, but not water, was withheld for 6 h prior to and for 2 h following drug administration. Water was available *ad libitum* throughout the study. The donkeys did not receive any medications for at least 2 weeks prior to commencement of the study and were acclimatized to the study environment during this period. At the completion of data collection, all donkeys were monitored for 1 week to ensure their health prior to returning to the entire herd.

### Study protocol

The study was approved by the Institutional Animal Care and Use Committee at Ross University School of Veterinary Medicine (IACUC #: 14-5-024 approved on 5/29/14). The donkeys were randomly assigned (http://www.random.org/lists) to receive each of the two treatments, intravenous butorphanol (IVB) and intramuscular butorphanol (IMB), in a crossover design, with at least 7 days of washout between treatments. Prior to drug administration and under aseptic conditions, a 14 gauge, 5.5” catheter (Abbocath, Hospira Inc., Lake Forest IL, USA) was percutaneously placed in each external jugular vein. Local anesthesia (up to 3 mL of 2% lidocaine hydrochloride) was placed subcutaneously in the area where the jugular catheter was placed. The right jugular vein catheter was used for drug administration, while the left jugular vein catheter was used for blood sample collection. Each donkey was weighed on an electronic scale immediately prior to drug administration for both treatment groups. The donkeys received an intravenous (IV) dose of butorphanol at 0.1 mg/kg (Torbugesic Injection; Fort Dodge Animal Health, Fort Dodge, IO 50501 USA), administered slowly over 1 min and flushed afterwards with 10 mL, 0.9% heparinized (2 units/mL) saline solution. For intramuscular (IM) administration, 0.1 mg/kg of butorphanol was given intramuscularly (18G × 1.5” needle) within the base of the right side of the neck. However, only one IV catheter in the left external jugular vein was placed when IMB treatments were given.

### Sample collection

For the IVB treatment: blood samples (5 mL each) were collected at time 0 (before drug administration) and at 3, 5, 10, 15, 30, and 45 min, and at 1, 2, 4, 8, 12, and 24 h. For the IMB treatment: blood samples (5 mL each) were collected at time 0, 5, 10, 15, 30, and 45 min, and at 1, 2, 4, 8, 12, 24, 36, and 48 h. The samples were collected by using a 3- syringe technique to avoid sample dilution. A blood volume of 10 mL was pulled from the left jugular catheter by using a 12 mL syringe containing 2 mL of heparinized saline. Then the blood sample was collected using a second empty sterile syringe. The blood contained in the first syringe was given back to the subject. Finally, a third syringe with 10 mL heparinized (2 units/mL) 0.9% saline solutions was used for flushing the left jugular catheter after collection of each blood sample. Catheters were removed either following flushing in the butorphanol (right side) or following collection of the 12-h sample (left side) and the remaining samples were collected by direct venipuncture (18G × 1.5” needle attached to a 12 mL syringe). Blood samples were collected into lithium heparin blood tubes (Monoject/Kendall; Tyco Healthcare Group, Mansfield, MA 02048, USA) and were centrifuged at 3,000 × *g* for 10 min. Plasma was immediately transferred into cryogenic vials (Corning Inc., Corning, NY 14831, USA) and stored at −80°C until analysis.

### Animal health monitoring

Routine health monitoring of the subjects including heart rate, respiratory rate, borborygmi (1–2-min auscultation of four quadrants) and rectal temperature were recorded at −15 and 0 min pre-drug administration and at 15, and 30 min, and 1, 2, 4, 8, 12, and 24 h post-drug administration for the IVB treatment and additionally for 36 and 48 h post-drug administration for the IMB treatment. Any adverse behavior (e.g., agitation, compulsive chewing, increased locomotor activity, ataxia, head jerking, nystagmus) or gastrointestinal tract effects (e.g., decreased borborygmi, decreased defecation, colic) were recorded at each sampling time.

### Sample analysis

Analysis of butorphanol in plasma samples was conducted using the Yarbrough et al. (24) method. The system consisted of a 2,695 separations module and a 2,475 fluorescence detector (Waters, Milford, MA, USA). Separation was attained on a Waters Symmetry C18 (4.6 × 75 mm, 5 μm) column with a Symmetry guard column. The mobile phase was a mixture of A: ammonium acetate buffer (0.05 M; pH 4.1), and B: acetonitrile. All solutions were prepared fresh daily filtered through a 0.22 μm filter and degassed before their use. The mixture was pumped at a starting condition of 77% A and 23% B for 2.7 min, then changed to 65% A and 35% B over 9.3 min and finally returned to initial conditions for the final 3 min. The flow rate was 1.3 mL/min. Fluorescence was measured at an excitation of 200 nm and an emission of 325 nm with the gain at 1000X. The column was maintained at an ambient temperature of 25°C.

Butorphanol was extracted from plasma samples using a liquid-liquid extraction. Previously frozen plasma samples were thawed and vortexed. One hundred microliters of plasma was transferred to a screw top test tube, followed by 10 μl of propylparaben (100 μg/mL internal standard). Two milliliters of ethyl acetate: hexane (40:60) was added. The tubes were vortexed for 1 min, and then centrifuged for 20 min at 1,700 × g. The organic layer was removed to a clean glass tube and evaporated to dryness with nitrogen. Samples were reconstituted in 225 μl of mobile phase. The solution was transferred to chromatography vials, and 100 μl was injected.

Standard curves for plasma analysis were prepared by spiking untreated plasma, from multiple individuals in the same herd of donkeys, with butorphanol which produced a linear concentration range from 5 to 1,500 ng/mL. The quality control samples used were 7, 300, 700 and 1,300 ng/mL. The acceptance criterion was ± 15% deviation from the nominal value except the LLOQ which was 20%. Intra-assay variability ranged from 1.7 to 8.4% and inter-assay variability ranged from 6.0 to 9.7%. The average recovery was 92%. The recovery of the internal standard was 97%. The lower limit of limit of quantification (LLOQ) was 5 ng/mL which represents a peak approximately five times baseline noise.

### Pharmacokinetic analysis

Plasma butorphanol concentrations were analyzed for each individual animal by compartmental and noncompartmental approaches using Phoenix 64 WinNonlin (Pharsight Corp., Mountain View, CA). Biexponential equations for a two-compartment model Cp = Ae^−α*t*^ + Be^−β*t*^ (where A and B are y-intercept constants, α is the rate constant of the distribution phase, and β is the rate constant of the elimination phase) were fit to the data. Weighting of the data using the reciprocal weighting of residual errors, using re-iterative fitting (i.e., 1/Yhat of the concentration) was used to improve the line fit and residual plots. The goodness of fit of the data with the model was determined by visual examination of the line fits, residual plots, and Akaike's information criteria (25). Values for elimination rate constant (λz), plasma half-life (t½), maximum plasma concentration (C_max_), time to maximum plasma concentration (T_max_), apparent volume of distribution (Vd_area_), apparent volume of distribution at steady state (Vd_ss_), total body clearance (Cl), and area under the plasma concentration time curve (AUC_0−∞_) from time 0 to infinity were calculated from non-compartmental analysis. While A, distribution intercept; B, elimination intercept; α, distribution constant; β, elimination constant; t½α, distribution half-life; t½ β, elimination half-life were calculated using a compartmental analysis. The AUC and AUMC were calculated using the log-linear trapezoidal rule. Mean residence time (MRT) was calculated as AUMC_0−∞_/AUC_0−∞_.

For butorphanol the extraction ratio (E_body_) was calculated as previously described (26, 27) with:


$$\begin{array}{l} {\text{E}}_{\text{body}}=\text{Systemic clearance/Cardiac output} \end{array}$$

First, calculations for each individual, and then combined for a mean value, with cardiac output described by Toutain et al. (26), as follows:


$$\begin{array}{l} \text{Cardiac output}=180\times {\text{BW(kg)}}^{-0.19} \end{array}$$

Parameter values were reported as the arithmetic mean of individually estimated parameters.

Variability in pharmacokinetic parameters was expressed as the standard deviation. In the case of the half-life, the harmonic mean and pseudo standard deviation were used instead.

## Results

### Animal health

No adverse behaviors (defined as agitation, compulsive chewing, increased locomotor activity, ataxia, head jerking, nystagmus) were noted throughout the study or for 48 h afterwards.

**Figure 1:**
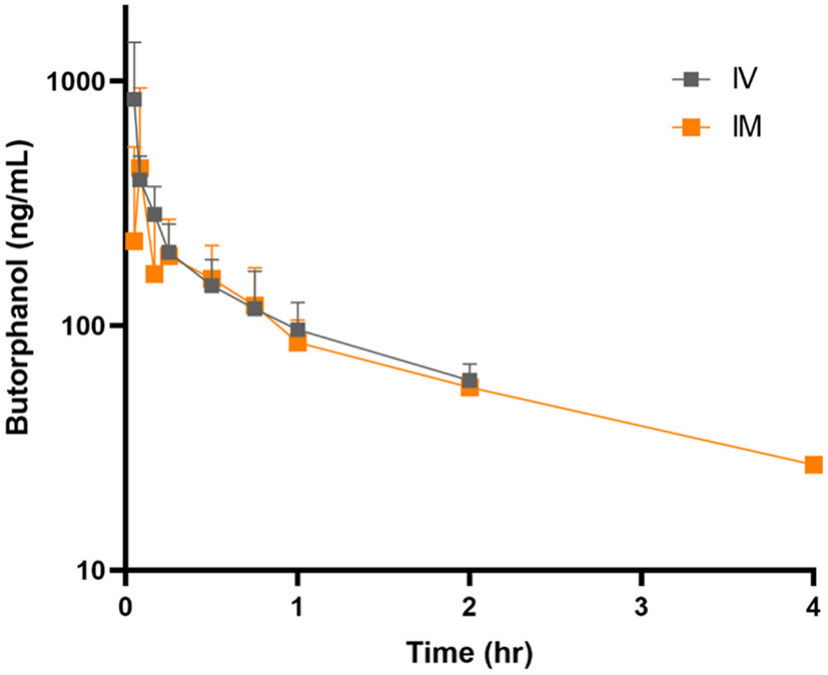
Mean ± SE plasma concentrations following intravenous (gray squares) and intramuscular (orange squares) administration of butorphanol (0.1 mg/kg) to donkeys.

Decreased borborygmi was noted in all subjects for both treatment groups for the first 1–2 h following administration of butorphanol but returned to baseline by the time the 4-h sample was obtained. The pharmacokinetic parameters after single IVB and IMB dosing are displayed in Table 1. Time vs. concentration curves are displayed in Figure 1 for IVB and IMB administration. Eight donkeys were originally enrolled in the study, but samples from 5 donkeys were used for the pharmacokinetic analysis due to loss of usable sample during international shipment.

**Table 1:** Plasma pharmacokinetic parameters of intravenous and intramuscular administered butorphanol (0.1 mg/kg) in donkeys.

**Pharmacokinetic parameter**	**IV (*n* = 5) mean ±SE**	**IM (*n* = 5) mean ±SE**	**IM No outlier (*n* = 4) mean ±SE**
T1/2 (h)^*^	0.83 ± 0.318	1.60 ± 0.397	1.71 ± 0.540
Elimination rate constant, λz (1/h)	1.34 ± 0.359	0.498 ± 0.089	0.482 ± 0.124
C0 (ng/mL)	3,028 ± 2,266	NA	NA
T_max_ (h)	NA	0.483 ± 0.093	0.563 ± 0.063
C_max_ (ng/mL)	NA	369 ± 190	180 ± 14
Cl (mL/h/kg)	400 ± 114	NA	NA
V_ss_ (mL/kg)	260 ± 45	NA	NA
V_d(area)_ (mL/kg)	322 ± 50	NA	NA
AUC_0−∞_ (h·ng/mL)	370 ± 131	410 ± 60	446 ± 68
MRT_0−∞_ (h)	0.83 ± 0.2	2.35 ± 0.67	2.68 ± 0.27
A (ng/mL)	662 ± 313	NA	NA
B (ng/mL)	267 ± 59	NA	NA
α (1/h)	23 ± 9.3	NA	NA
β (1/h) t½ α (h) t½ β (h) E_body_ (%) F	1.05 ± 0.21 0.054 ± 0.018 0.796 ± 0.181 5.64 ± 1.58	NA NA NA NA 1.33 ±0.45	NA NA NA NA 1.59 ± 0.47

* Harmonic mean.

Vd_area_, Volume of distribution; Vd_ss_, apparent volume of distribution at steady-state; Cl, total body clearance; λz, elimination rate constant; ^½^, plasma half-life; AUC_0−∞_, area under the plasma concentration time curve from time 0 to infinity; MRT, mean residence time; A, distribution intercept; B, elimination intercept; α, distribution constant; β, elimination constant; t½ α, distribution half-life; t½ β, elimination half-life; E_body_, Extraction ratio; NA, not applicable.

**Figure 2:**
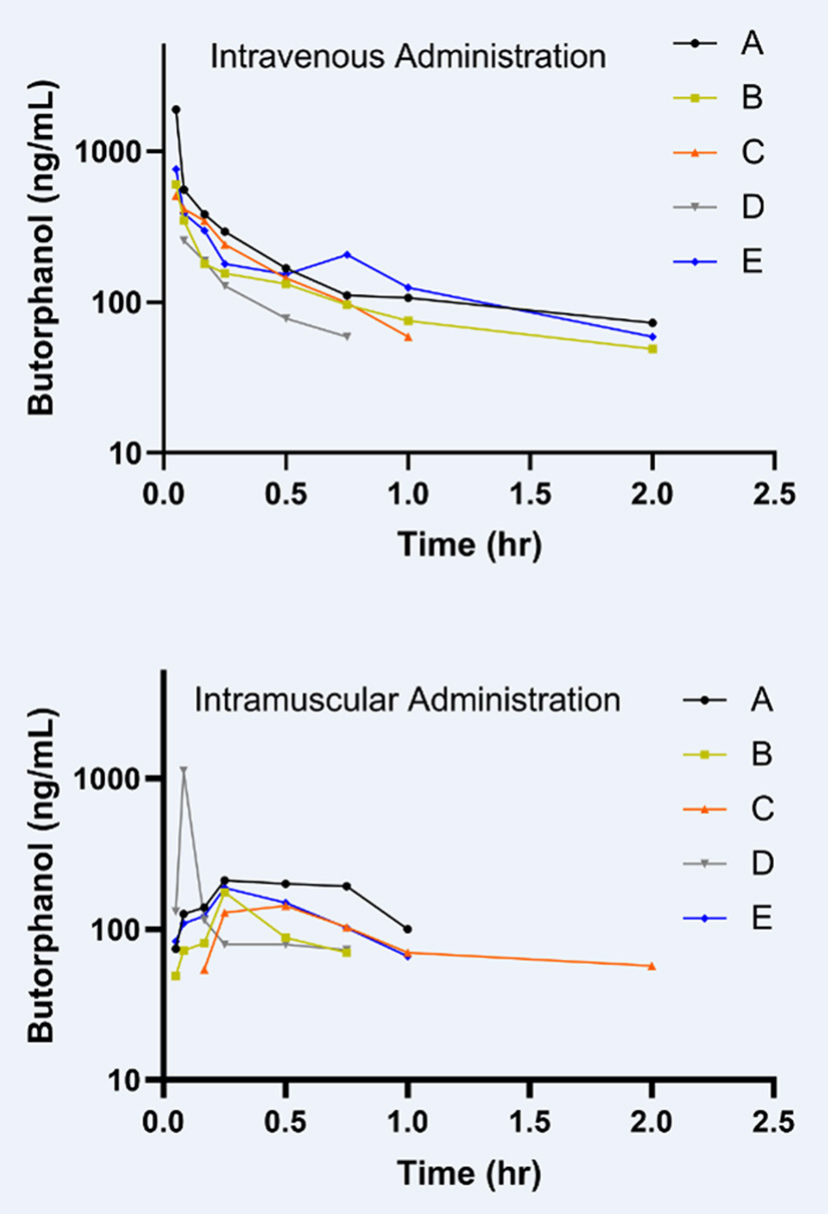
Individual plasma concentration time curves of each of the five animals (A–E) for the IV butorphanol and IM butorphanol treatment groups.

The intramuscular group is presented with and without a donkey that appeared to have an outlier plasma concentration in the initial sampling period as seen in Figure 2. Data presented as mean ± standard error.

## Discussion

To the best of the authors' knowledge, this is the first report on the pharmacokinetics of butorphanol in donkeys. The results of this investigation in donkeys demonstrates several differences from the pharmacokinetics of butorphanol previously reported in horses. Other studies have evaluated the pharmacokinetics of intravenously administered butorphanol in horses (13, 14, 16–18). The plasma clearance reported in this study of donkeys (400 mL/kg/h) is less than reported in a population of seven Thoroughbred geldings (646 mL/kg/h) (18), ten horses of different breeds (650 mL/kg/h) (16), a population of ten Thoroughbreds (690 mL/kg/h) (13), as well as a study of six Arabian-pony cross foals (1,882 ± 261 mL/kg/h) (17). The reported extraction ratio of 5.64% would be considered a low overall body extraction ratio (26). The Cmax in these donkeys, 369 ± 190 ng/mL, was higher than the reported Cmax of 99.2 ± 29.5 ng/mL in adult horses that received a lower dose of 0.08 mg/kg butorphanol IM (15). Interestingly, the horses that received a lower IM dose of butorphanol had a shorter Tmax of 0.32 h compared to the donkeys with a Tmax of 0.48 h after IM butorphanol administration. As seen in Figure 2, there is one donkey that had an unexpected concentration time curve making it most likely an outlier due to a potential analytical lab labeling error. Analysis of the remaining four donkeys resulted in a Cmax of 180 ±14 ng/mL which is lower than when all five donkeys are considered but still higher than reported Cmax in horses. The Tmax when the outlier is excluded from the IM butorphanol is 0.56 h and the AUC increases from 410 ± 60 to 446 ± 68 h·ng/mL. These changes in PK parameters when the outlier donkey in the IM butorphanol group is excluded should be taken into consideration for future studies.

The plasma half-life (T1/2) reported in these donkeys after IVB administration of 0.83 h was shorter than the ranges of 2.31–7.77 h reported in adult horses (13, 14, 16). T1/2 after IMB administration of 1.60 h was longer than that noted in horses of 0.57 h (15). In horses, the longer elimination after IV vs. IM administration was attributed to a deep compartment after IV administration in horses that was not detected after IM administration (16). This deep compartment was not observed in the donkeys in our study. An explanation for the difference between half-lives after IV and IM administration in these donkeys could be flip-flop kinetics where elimination and absorption overlap or the rate of drug absorption is slower than the rate of elimination. The inter-individual variations in pharmacokinetic parameters (T1/2, MRT, AUC) between the two treatment groups as seen in Table 1 were not statistically different between groups. Pharmacokinetic parameters can be influenced by assay sensitivity, with lower limits of detection and quantification extending reported parameters such as elimination half-life (28). This could explain the differences in parameters reported in the donkeys in this study when compared to horses as this study's method had a limit of detection of 5 ng/mL compared to 0.1 ng/mL for several reported equine studies (13–16). However, analytical sensitivity would not impact parameters such as Cmax and Tmax.

The bioavailability of butorphanol after IM administration to horses has been described as 37.3% (15) and 87% after subcutaneous administration (16). The mean ± standard error IM bioavailability in the five donkeys in this study was approximately 133 ± 45%. While this could be suggestive of a species-specific difference in bioavailability between horses and donkeys, it could also be an artifact of the observed areas under the curve. Bioavailability can be affected by a number of parameters within individuals. Factors such as drug concentration at the site of administration, absorption site surface area, the pKa of a drug, the molecular size of a drug, and the pH of the fluid surrounding the site of drug administration could all affect the bioavailability (29).

The authors can only speculate that one of these factors might be the cause of the difference seen or there may be alterations in muscle blood flow in these individuals that could account for the changes seen. A more likely cause was that an insufficient number of early samples were obtained to adequately describe the absorption phase. Alternatively, it could be due to inter-individual variability in the rate of absorption as some individuals did not adhere to expected rise and fall from the IM administration. A bioavailability of >100% for morphine has been reported in goats (30), llamas (31), and dogs (32) and this could be attributed to a limited or minimal distribution phase.

Limitations of this study include the small sample size. However, for veterinary pharmacokinetic studies, a sample size of 4 to 6 animals typically is sufficient to describe the pharmacokinetics of a test drug (33). The authors recognize that most pharmacokinetic investigations in other species studied at least 6 animals. The data in this study is being presented as a preliminary study because of the small sample size that was able to be evaluated in a cross-over manner.

Future studies of the pharmacokinetics of butorphanol in donkeys should consider higher doses, pharmacodynamic effects, and increased number of sampling timepoints to more reliably determine bioavailability. Additional work could involve utilizing nonlinear mixed-effects modeling to determine factors for the variation of pharmacodynamics of butorphanol in the donkey population (34). Determining the effective dose of butorphanol in donkeys would benefit those working with this species in clinical practice.

In conclusion, in this preliminary study, administration of 0.1 mg/kg butorphanol, by both the IM and IV route, to healthy adult donkey geldings is characterized by a rapid elimination half-life.

Higher peak concentrations were achieved after IVB administration. However, butorphanol was detectable for longer after IM administration (4 vs. 2 h).

## Data availability statement

The original contributions presented in the study are included in the article/supplementary material, further inquiries can be directed to the corresponding author.

## Ethics statement

The animal study was reviewed and approved by Ross University Institutional Animal Care and Use Committee.

## Author contributions

LE, OO, BS, and IL contributed to study design and implementation. SC developed the analytical method and performed the pharmacokinetic analysis. JS contributed to analysis and results interpretation. All authors contributed to manuscript construction.

## Funding

This project was funded by a Ross University School of Veterinary Medicine intramural grant. The funder was not involved in the study design, collection, analysis, interpretation of data, the writing of this article or the decision to submit it for publication.

## Conflict of interest

The authors declare that the research was conducted in the absence of any commercial or financial relationships that could be construed as a potential conflict of interest.

## Publisher's note

All claims expressed in this article are solely those of the authors and do not necessarily represent those of their affiliated organizations, or those of the publisher, the editors and the reviewers. Any product that may be evaluated in this article, or claim that may be made by its manufacturer, is not guaranteed or endorsed by the publisher.
